# Analysis of over 1600 chemistry YouTube channels from 2005 to 2023

**DOI:** 10.1098/rsos.241599

**Published:** 2025-01-29

**Authors:** Scott Gardner, Gabriela Bezati, Tristen Godfrey, Katie Baird, Usamah Bilal, Emma Loudon, Rhona Young, Lewis E. MacKenzie

**Affiliations:** ^1^ Department of Pure and Applied Chemistry, University of Strathclyde, Glasgow, Scotland G1 1RD, UK; ^2^ Department of Chemistry, Princeton University, Princeton, NJ 08544, USA

**Keywords:** science communication, youtube, chemistry, new media, quantitative analysis, emerging media

## Abstract

Chemistry has found broad appeal on the freely available global video-sharing platform YouTube, with some YouTube videos even being cited in the peer-reviewed chemistry literature. By applying both manual and semi-automated search methods, we identified, categorized and analysed publicly available data for 1619 chemistry YouTube channels that were available in 2023. Forty-nine per cent of channels were active in 12 months prior to sampling. The majority of channels were produced by independent content creators with no clear affiliation or background and 71% of channels were for the purposes of learning or exam revision. YouTube video production spiked in 2020, coincident with the COVID-19 global pandemic. We also examined the number of videos produced, channel lifespans, the use of features such as playlists and short-form videos, apparent revenue streams (outside of default advertising), the use of other social media and whether or not channels were exclusively producing chemistry content. This study and the associated dataset provide the first large-scale ‘census’ of how YouTube is being used for chemistry communication and education worldwide. We expect our findings to be of interest and use to policy makers, funding agencies, educators, content creators and the public.

## 1. Introduction

The advent of high-speed internet means that science, technology, engineering and mathematics (STEM) topics are no longer confined to school classrooms, university lecture theatres or textbooks [1]. Rather, a wealth of STEM information is readily available through a simple internet search: from early-learning resources for children, right through to long-form scientific protocols. Indeed, a wide variety of ‘new media’ has emerged to meet the public appetite for science, in the forms of social media [2], text-based blogs [3], audio podcasts [4] and videos [5,6]. Each medium has its own advantages and disadvantages, and, consequently, STEM subjects are not equitably represented across these different platforms. For example, in our 2019 large-scale study of science podcasts, we found that chemistry is underrepresented in the medium of audio-only podcasts when compared to biosciences and physics [3]. Notably, however, chemistry has found widespread appeal and popularity in video format, where the visual features of chemistry—colour changes, energetic reactions, unusual apparatus, fumes, bubbles, etc.—make for appealing audiovisual content [7].

Since 2005 [8] YouTube has emerged as the leading website for sharing video content, being the second-most visited site on the World Wide Web, with over 1.9 billion users in 2019 and 2.41 billion users in 2023 [9,10]. YouTube is a free-to-use, user-driven video streaming platform, available in the majority of countries in the world and is accessible through computers, mobile devices, games consoles and smart TVs. YouTube’s primary function is that of entertainment, but it is also an excellent tool for global communication, making it an ideal platform for education as well. The typical YouTube video is intended to attract a large audience, with a relatively short duration (typically between 5 and 30 min), and an engaging manner of presentation. Content creators can set their content to private, public or ‘unlisted’ (only accessible to those with the URL). Furthermore, viewers can interact with content creators and the wider associated community through methods, such as video comments, audience polls and social media. A myriad of content can be found on YouTube, ranging from low-quality videos by amateurs to high-end productions by professional creators (referred to as ‘YouTubers’) who earn enough advertising revenue to make a living from their contributions to the platform.

There is a wide range of chemistry content on YouTube, from experiments for children (reminiscent of classic chemistry ‘kits’), to school-level demonstrations, to university exam revision materials, to advanced postgraduate and postdoctoral level syntheses based upon peer-reviewed literature. In fact, YouTube videos covering chemical synthesis procedures have even begun to be referenced in contemporary peer-reviewed scientific literature [11,12], demonstrating that video content is not only engaging and entertaining, but also useful for disseminating cutting-edge scientific research.

The wider popularity of chemistry on YouTube is evidenced by the subscriber count of a channel^1^ [8]. A variety of engaging channels that showcase chemistry content as a form of entertainment as much as education, i.e. ‘edutainment’, have a large number of subscribers [13,14]. An excellent example of a multi-disciplinary science edutainment channel is *CrashCourse*, with over 15.7 million subscribers and 1558 videos covering a wide range of subjects [15]. More specific to chemistry, the channel *NileRed* is a prominent example, with over 7.6 million subscribers and 348 videos at the time of writing (November 2024) [16]. *NileRed* typically covers long-form (up to 1 h) complex multi-step synthetic chemistry procedures filmed in a private laboratory space. The videos include in-depth usage of synthesis apparatus and often detail where synthesis procedures fail as much as when they succeed, aptly demonstrating the trial-and-error nature of the scientific method [17], as well as showing that each video can be a learning process for the creator too [18]. Despite their length and in-depth nature, a typical *NileRed* chemistry video is engaging and designed to attract viewers, with titles such as *Turning plastic gloves into hot sauc*e (1 h 8 min) [19 million views] [19], *Making transparent wood* (44 min) [21 million views] [20], *Making the stinkiest chemical known to man* (44 min) [14 million views] [21], *Making a deadly chemical in my parent’s garage* (34 min) [6.9 million views] [22] and *Does cyanide actually smell like almonds*? (22 min) [5.9 million views] [23]. A variety of other channels produce similarly sophisticated and complex content, with some notable examples of high-level scientific trends emerging. For example, channels such as *Extractions&Ire* (>2 13 000 subscribers) [157 videos] [24] and *Chemiolis* (>1 30 000 subscribers) [69 videos] [25] competed to be the first channel to produce the compound cubane [26] through multi-step organic synthesis procedures outside of a professional laboratory setting [27,28]. This ‘cubane race’ meme [29] drove casual audiences’ engagement with advanced organic chemistry, with other channels subsequently producing related content, such as *That Chemist* (>2 59 000 subscribers) [233 videos] [30] producing a video covering the history of cubane and cubane derivatives [31]. While this could be glibly regarded as a trivial example of an internet meme [32], the cubane race is notable because it provided the general public with an insight into synthetic organic chemistry (otherwise generally confined to university-level classes) to create a form of community-driven science edutainment. In a 2024 video, *Periodic Videos* (>1.61 million subscribers) [725 videos] [33], based at the University of Nottingham, noted that there are university graduate-level chemistry students who have grown up watching chemistry YouTube channels [34]. It seems that YouTube is emerging as a new, accessible and engaging form of science capital [1], serving as the ‘shop window’ for chemistry, where viewers are exposed to concepts and techniques that would otherwise be the purview of selective tertiary education or intimidating and hard-to-access textbooks.

There are many YouTube channels more specifically dedicated to the tutorial/assessment/revision aspects of education; two prominent examples being the multi-disciplinary *Khan Academy* (>8.6 million subscribers) [>8600 videos] [35] and *The Organic Chemistry Tutor* (>8.6 million subscribers) [>2800 videos] [36]. Likewise, individual educators have also taken to setting up their own channels with tutorial and revision-style content, particularly during the COVID-19 pandemic, during which in-person teaching was restricted. Some examples include *Mike Sugiyama Jones* (>46 000 subscribers) [972 videos] [37] and *Professor Derricotte* (>15 000 subscribers) [232 videos] [38]. The typical uploads of these types of channels are similar to that of a lecture, covering material that is often found in high-school or university curricula. However, each topic is typically broken down into shorter-form videos (~10 min in length) functioning as segments of full lectures, and organized into playlists by topic, allowing for convenient navigation. In contrast to traditional education and revision sources—such as textbooks and tutors—these channels function as a freely accessible learning resource, and as such, represent a fundamental shift in how students can engage in science education and learning.

The potentially hazardous nature of chemistry is an important concern in chemistry YouTube videos, both for reasons of public safety, and for the YouTube advertising income which may support a channel. If a video is perceived as showcasing dangerous content or otherwise violating YouTube’s community guidelines, the advertising revenue associated with the video may be withheld from the creator (‘demonetization’) [39,40], or more severely, the channel may be removed from YouTube altogether. This means that content creators risk having all their work permanently expunged from the platform, potentially even more so than their counterparts in other fields [41]. Furthermore, chemistry content creators may be under the auspices of national authorities regarding chemical substances. For example, the channel *styropyro* (>2.6 million subscribers) [199 videos] [42] reported a visit from legal authorities in the United States regarding purchasing chemical supplies in large quantities for use as reagents in the channel’s videos [43]. As another example, the creator of *NileRed* also reported contact from Canadian national authorities regarding large-scale chemical equipment purchases for their channel [44]. To counterpoint this, YouTube can also be used to highlight safety in chemistry. Some chemistry YouTube channels even make safety concerns a point of interest, such as the channel *That Chemist*, with videos titled ‘*Safety isn’t Optional—PPE Tierlist*’ (21 min) [>1 50 000 views] [45], ‘*Which Chemical Is The Worst Carcinogen?’* (44 min) [>13 00 000 views] [46] and ‘*Which Gases are the Most Toxic?*’ (22 min) [>4 34 000 views] [47]. While such safety-orientated humour may seem trivial, they are also a form of informative edutainment. Ultimately, chemistry YouTube channels are setting an example to people who view their content, so communication of chemistry safety is vital.

Safety concerns highlight the fact that it is particularly important that chemistry content is produced by suitably qualified individuals or teams capable of handling chemicals and undertaking experiments in a safe manner. Some chemistry YouTube channels display their associations openly, for example, *Periodic Videos* has been produced by professional chemists at the University of Nottingham since 2008 [48], with the long-running format enabling viewers to gain familiarity with the creators. It is important to note that members of the public tend to trust and engage with creators if they can gain familiarity with the people making the content [49–51]. However, many chemistry YouTube channels appear to be made by independent science enthusiasts with no clear backing from established scientific or media organizations. Indeed, prior research has shown that user-generated content has a popularity edge over professionally produced content [8]. Therefore, there is a clear divide between channels with identified and authoritative content creators, and those without clear attribution to individuals, educational institutions, or other organizations.

Given the popularity of YouTube as a freely available form of science capital [1], and that chemistry underpins modern society and humanity’s response to climate change [52], it is important to gain a large-scale overview of what chemistry content is being made, by whom and for what audience(s).

The aim of this research is to provide the first large-scale systematic ‘census’ of chemistry YouTube channels and their associated content in order to better understand the importance of YouTube and social media within the wider ecosystem of global science communication and education.

## 2. Methods

### 2.1. Methodology overview

To comprehensively identify YouTube channels publishing chemistry content, both manual and semi-automated searches were undertaken (§2.2) followed by data categorization/coding, validation and analysis (§2.4 and §2.5). This methodology was developed in consultation with the University of Strathclyde Department of Pure and Applied Chemistry Ethics Committee and the central University of Strathclyde Ethics Committee. All data gathered and examined were extracted from publicly online material directly relating to the YouTube channels; this could be broadly described as the sort of information a dedicated viewer of the channel may find online (e.g. through YouTube, websites and other social media for each channel). The channel search and initial data categorization/coding was undertaken between 20 June 2023 and 1 August 2023 by two undergraduate chemistry student operators, G. Bezati and T. Godfrey, under the supervision of L. MacKenzie. This gave a total of three operators for channel search and initial data categorization/coding. Subsequent detailed data validation, reclassification and error-checking were undertaken manually by S. Gardner between 8 February 2024 and 17 May 2024, with supervisory input from L. MacKenzie. All work was conducted using personal laptop computers running Google Chrome in standard mode. Data for each channel identified were collated into a secure cloud-based spreadsheet (Google Sheets). Data analysis was undertaken using Google Sheets and Microsoft Excel.

### 2.2. Identification of candidate channels

#### 2.2.1. Manual search and validation

**Figure 1.**
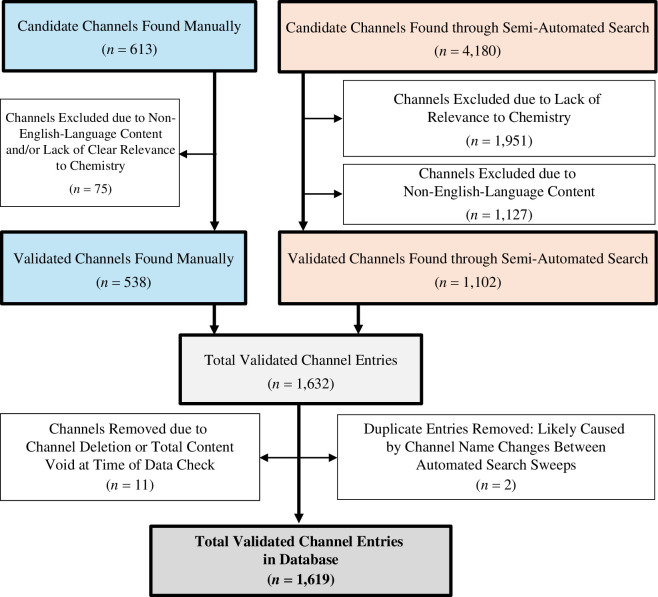
Summary of the channel identification, validation and verification process.

A manual search was first undertaken through the YouTube search function based upon (1) a preliminary list of known chemistry YouTube channel names and (2) searching the keywords ‘chemistry’ and ‘all about chemistry’ and taking the first 20 results for each. (3) Channels were also found when they were (a) linked in other YouTube channels’ ‘about’ pages or (b) recommended by the YouTube recommendation system. From this YouTube recommendation approach, each channel would typically result in the suggestion of several other channels (for instance, over 100 candidate channels were identified in the span of 2 days from recommendations alone). Consequently, further manual keyword searches were not required. Channels were then validated by the operators by applying inclusion criteria (§2.3). An overview of manual search results and the following exclusions is shown in figure 1.

#### 2.2.2. Semi-automated search and validation

To follow on from the manual search through semi-automated methods, the operators manually generated a list of 236 chemistry-related keywords (electronic supplementary material, table S1). A Google Apps script was developed to automatically conduct a YouTube search for each keyword, and then compile the names of videos and associated channel names for the top 50 videos results for each keyword (code provided as electronic supplementary material). This information was then compiled into a spreadsheet. Duplicate results were cross-referenced and were manually verified to filter out irrelevant results and ensure that duplicates removed from the database were in fact duplicate entries of the same channel, and not entries of different channels with duplicate names. The semi-automated search concluded after running through the list of keywords. This number of keywords was found to be satisfactory since, as the search progressed, more and more channels kept appearing in duplicate on the list. This indicated that the search was near exhaustive, and the addition of new keywords would not bring up many new channels.

Any channels found by both the manual and automatic search methods are listed in the database as being found manually, due to the fact that the manual search occurred prior to the automatic search.

### 2.3. Inclusion criteria

The following criteria were developed and applied with the aim of ensuring that only YouTube channels publishing valid chemistry content were included for analysis.

#### 2.3.1. Inclusion criteria A: English language channel content

Only English-language YouTube channels were included for analysis. This limitation derives from English being the common language of study authors and a widely used international language for science communication [53]. To verify that a channel was presenting content in English, between three and five videos from a candidate channel were randomly sampled and manually viewed. This manual verification was necessary because some channels may have had English-language names or video titles while the audiovisual content was in another language.

#### 2.3.2. Inclusion criteria B: Chemistry-related channel content

In this study, we defined a chemistry YouTube channel as a YouTube channel which produces videos on the topic of chemistry, either exclusively or as a subset of its total video content.

Therefore, to be included in this study, candidate YouTube channels must have uploaded one or more publicly accessible videos that were directly related to chemistry, regardless of other topics covered by the channel. Candidate channels whose content was not relevant to chemistry or otherwise unclear were excluded. For practical reasons, the chemistry content had to have been readily apparent, i.e. present in a large proportion, categorized into a playlist, or referred to in the channel’s ‘about’ section.

### 2.4. Data harvesting

#### 2.4.1. Semi-automated quantitative data collection

Publicly available quantitative metadata for each channel was collected using a Python program (see electronic supplementary material for code). The information collected through this method was channel name, channel ID, subscriber count, total number of videos, total number of views, channel creation date, date of first video upload, date of latest video upload, total content duration, country associated with the channel and the date that all of the above data were collected (table 1).

**Table 1.** Automatically collected channel information.

category	definition
**channel name**	the display name of the channel at the time of sampling
**channel ID**	the unique identifier code for the channel. Every YouTube channel’s ID is automatically generated and assigned by YouTube when the channel is created. This was found for the manually sourced channels upon identification using an online YouTube Channel ID Finder [54]
**subscribers**	the subscriber count of the channel at the time of sampling. A subscriber is a user account which has opted in to receive content updates from a channel, therefore the subscriber count is a good measure of a channel’s popularity
**total videos**	the total number of publicly available videos that a channel had uploaded at the time of sampling
**total views**	the total number of views across all of the channel’s videos at the time of sampling
**creation date**	the date on which the channel was created
**date of first video**	the date that the oldest available video on the channel was uploaded
**date of latest video**	the date that the latest video on the channel was uploaded (at the time of sampling)
**total content duration**	the sum of the length of all of the channel’s videos
**country**	the country in which the channel has declared it is based
**manual/automated data collection**	whether the channel was sampled manually by the operators or automatically by the semi-automated search algorithm. In cases of channels being found by both means, they have been marked as being sampled manually
**automated data collection date**	the date that the channel was found by the semi-automated search algorithm

#### 2.4.2. Manual data categorization and coding

The following data were coded (subjectively interpreted) by multiple operators for each channel: channel affiliation, profit/non-profit status, active status, creator background, perceived target audience and content genre(s) (table 2). Channel URL, multi-disciplinary content, playlist categorization, proportion of chemistry content, use of YouTube ‘Shorts’ feature, use of YouTube ‘livestreams’ feature, additional revenue sources and links to other websites/other social media platforms were found manually, but did not require coding by the operators due to their objective nature (table 3). Where the two primary coders were unable to determine categorization, a third coder was consulted and the majority view was taken. Only information which was publicly available at the time of data collection was used. The information utilized was exclusively obtained from the channel description, video titles or descriptions and web links provided on the channel because these are regarded as primary sources of public information uploaded by the channel creators. Extraneous third-party sources (e.g. video interviews with content creators) were not utilized for matters of practicality.

**Table 2.** Manually collected channel information—coded.

category	definition and examples	options
**affiliation**	What type of entity the channel was associated with (if any). **—Charity/Non-profit**. Organizations which have declared themselves as such. For example, *Imagination Station Toledo,* associated with a science museum in Ohio, USA [55]; and *ChemTalk*, a non-profit organization providing chemistry learning resources for free [56]. **—Corporate**. A commercial entity. For example, *FlinnScientific* [57] and *Thermo Scientific Spectroscopy & Materials Analysis* [58]. **—Independent**. A channel which had no affiliation. For example, *NileRed* [14]. **—Learned society**. Channels associated with a professional scientific body or learned society. For example, The official channel of the *Royal Society of Chemistry* (RSC) [59] and the channel *Reactions*, which was created by the American Chemical Society (ACS) [60]. **—Private education provider**. Content was structured in a course format, irrespective of any recognized qualification pathway or pay-to-access features. For example, *Khan Academy* or small tutorial companies such as *Science with Hazel* [61]. **—School**. Associated with a primary or secondary level educational institute, such as a primary school or high school. For example, *North Carolina School of Science and Mathematics,* a high school in the USA [62], and *Malmesbury Education*, a secondary school in England [63]. **—University/College**. Associated with a tertiary-level education institute, such as a university or college. For example, *Periodic Videos* is associated with chemistry at The University of Nottingham, and *UCI Open* was created by The University of California, Irvine to publish course materials [64].	see definitions ←
**profit/non-profit**	**Profit**—The channel was either a corporate entity which had not declared themselves ‘non-profit’, and/or they sell products or services on their affiliated website. **Non-profit**—The channel’s affiliated entity declared themselves as such in the channel’s about page, on their website, or on an associated profile.	[profit], [non-profit], [undeclared], [N/A]
**active status**	**Recently active**. The most recent video was uploaded <3 months prior to the sampling date. **Dormant**. The most recent video was uploaded 3–12 months prior to the sampling date. **Inactive**. The most recent video was uploaded >12 months prior to the sampling date.	see definitions ←
**creator(s) background(s**)	the background of the person, people or group(s) making and uploading videos on a channel. Channels may have had multiple creator backgrounds reflecting individual or team experiences. **—Amateur**. Channel creators self-declared that they do not have any qualifications or professional experience relating to chemistry or associated disciplines. For example, *Brainiac75* declares themselves as an amateur [65]. **—Educator(s**). Channel creators were schoolteachers, school tutors, university or college lecturers, professors, etc. For example, Nick Duell of *Duell Chemistry* is a teacher at a high school in Massachusetts, USA [66], and Prof. Sir Martyn Poliakoff of *Periodic Videos* is a professor at The University of Nottingham [67]. **—Institution(s**). The channel was created by one or more learned societies, professional scientific bodies, universities, colleges or schools. For example, *The Royal Institution* has published a wide range of chemistry content [68], and there are several channels associated with the Indian National Programme on Technology Enhanced Learning (NPTEL), such as *nptelhrd* [69] and *NPTEL-NOC IITM* [70]. **—Journalist(s**). A channel creator was a journalist. For example, Brady Haran of *Periodic Videos,* or *Hamilton Morris* [71]. **—Organization**. Any company or other group of people/institutions structured together. For example, *Khan Academy* is a non-profit private education provider (see ‘affiliation’ category), but it is classed as an institution because it is not an accredited place of learning. **—Professional science communicator(s**). Channel creators were dedicated to educating the general public about science, likely in a professional capacity. For example, Steve Spangler of *Sick Science!* [72] and *SpanglerScienceTV* [73]. **—Professional Scientist(s)/Engineer(s)/Medic(s**). Channel creators had declared themselves to be a scientist, engineer, or medic. For example, Ben Krasnow of *Applied Science* [74], Dr Josiah Newton of *That Chemist* and *Dr Jessica Gomez* [75]. **—Professional YouTuber(s**). Channel creators were full-time YouTube content creators and may have multiple distinct channels across several themes. For example, *CrashCourse* was created by professional YouTubers Hank Green and John Green, who are producers of multiple channels, including *vlogbrothers* [76 [. **—Student(s**). Channel creators were school or university/college students. For example, the creator of *Chemdelic* states that they are currently a chemistry student [77], and *chatzida* was created in part by high school student [78].	see definitions ←
**perceived target audience**	the demographic to which a channel’s content was ostensibly aimed towards. **—Children**. The topics covered appealed to children. The videos produced by the channel covered basic topics, often using bright visual aids, simple language and perhaps songs to reinforce learning points and emphasize fun. **—General public**. The channel content was intended for adults with minimal chemistry/general science knowledge. The videos were presented in a way that was easy for the average person to understand. **—Professionals**. The channel content appeared to be directed towards scientific professionals in chemistry or a related field. The topics covered were usually quite complex due to the videos being intended for people who have a firm grasp on basic and intermediate topics already. One common type of video was laboratory instrument demonstrations. **—Students**. The channel’s content was directed towards student learners in primary, secondary or tertiary education. Videos were often presented ‘lecture style’, akin to the experience of being in a classroom or a lecture hall and may focus on pertinent topics, such as how to pass various examinations.	see definitions ←
**content genre(s**)	the general type(s) of video content that a channel uploaded. A channel could span multiple genres. **—‘Backyard’/Home Chemistry**. Videos featuring simple experiments or demonstrations. The common theme was that the procedures were usually performed outside or at home without professional equipment. A recurring theme of such channels was that content was often designed for ‘shock and awe’, e.g. freezing household items in liquid nitrogen, dissolving items in acid, or generating explosions. **—Children’s**. Any sort of content that appeared to appeal to young children. Videos often covered simple topics and often rely on colourful graphics or music. **—Experiments/lab demonstrations**. Videos showcasing reactions or lab techniques, filmed in a laboratory setting. **—Promotional material**. Videos designed to promote the entity a channel is affiliated with, usually an organization or institution. For example, product advertisements/demonstrations or university tours. **—Revision/theory:** Videos covering any type of chemistry theory. The videos may have covered a specific topic or module and been structured in a manner similar to a lecture, but this was not a requirement. **—Scientific novelties and curiosities**. Videos showcasing interesting objects or concepts. **—Seminars/Conferences/Interviews**. Videos featuring short excerpts or full-length recordings of seminars, conference symposiums or interviews. **—Software**. Videos focusing on software related to chemistry. **—Vlogs**. Informal video logs, often more casual in presentation. For example, videos documenting scientific site visits or ‘A Day in the Life of a Chemistry Student’	see definitions ←

**Table 3.** **Manually collected channel information**.

category	definition	options
**URL**	the web address of the channel	URL
**channel rename (at time of data check**)	the new name of the channel, if the name had changed between initial sampling and data checking	
**multi-disciplinary**	channels were deemed to be multi-disciplinary if <95% of videos were associated with chemistry content. An exception was made for ‘meta’ content, such as channel update videos. Any channel with ≥95% chemistry content was deemed to be ‘chemistry only’	[Multi-Disciplinary], [Chemistry Only]
**playlist categorization**	whether or not the channel utilized YouTube playlists to organize content	[Yes], [No]
**dedicated chemistry playlist(s**)	whether or not the chemistry content on the channel was organized into its own playlist(s). Only applicable to multi-disciplinary channels with playlists	[Yes], [No], [N/A]
**estimated chemistry content proportion**	the estimated percentage of total channel content that was related to chemistry. N.B. For channels that were exclusively chemistry (or near to), these were categorized as >95%	approximate number
**‘shorts’**	whether or not the channel had posted ‘Shorts’, a video format defined by YouTube as vertical videos of 60 s or less [79].	[Yes], [No]
**livestreams**	whether or not the channel had hosted livestreamed videos on YouTube	[Yes], [No]
**additional revenue**	whether or not the channel advertised sources of income (other than YouTube advertisement revenue). Definitions: **—Commissions/Sponsors**. The creator has accepted funding from another individual or organization to create specific content. **—Donations**. A link to another webpage through which the user can donate money to support the content creator. **—Educational resources**. A link to another webpage where users can purchase or subscribe to related learning content. **—Merchandize**. A link to an online shop selling channel-branded products, such as clothing. **—Other streaming platforms**. Link(s) to associated profiles on other video streaming platforms, such as Twitch or Weibo. **—YouTube membership**. Channel memberships allow users to support the channel through monthly payments, often receiving exclusive perks in return.	see definitions.
**website**	the link to a channel’s dedicated website*	URL
**patreon**	the link to a channel’s associated Patreon profile*	URL
**X**	the link to a channel’s associated X profile (A.K.A. Twitter before July 2023)*	URL
**Facebook**	the link to a channel’s associated Facebook page*	URL
**Instagram**	the link to a channel’s associated Instagram profile*	URL
**TikTok**	the link to a channel’s associated TikTok profile *	URL
**email**	a channel’s associated email address. *	URL
**other URL(s**)	he link(s) to any other website(s) *	URL
**sampling date**	the date the channel entry was added to the database	
**data check date**	the date the channel’s data was verified	

An asterisk (*) denotes information was gleanedfrom links in a YouTube channel’s ‘about’ page.

The proportion of chemistry content of channels with a manageable number of video uploads was calculated by individually counting the number of chemistry videos. The proportion of chemistry content of channels with large numbers of uploads was estimated by random manual sub-sampling of videos. For channels including dedicated chemistry playlists, the number of uploads in chemistry-related playlists was used to quantify overall chemistry content.

### 2.5. Data checking and reclassification

After the database was created, data checking and reclassification were performed to both verify and improve the clarity of results. This involved manually parsing each entry in the database and comparing it with the channel page on YouTube to identify any inconsistencies and/or errors, including any duplicate entries or channels with a total content void. At this stage, the URLs for each channel were manually added, since this did not occur during the initial sampling. Several data categories were added or reworked for clarity and consistency (electronic supplementary material). Any channel name changes made since initial sampling were also noted in a separate column. Following these steps, the estimated chemistry content proportion was checked for around 20 of the top 100 channels (in order of subscriber count) and around another 30 randomly selected channels to ensure the original estimate was accurate, and to provide an estimate for every channel in the top 100. Several aesthetic changes were made during this process as well, including formatting of cells to a uniform size, renaming of data columns and classifications and spell-checking. The date that each database entry was verified was also recorded.

Data were preserved as far as possible from the initial sampling date, i.e. the subscriber counts, total videos, etc., were not updated at the time of the data check.

## 3. Results

The channel identification, validation and verification process is summarized in figure 1. Six hundered and thirteen candidate channels were identified through the manual search method, with 75 of these excluded after applying inclusion criteria (§2.3). A total of 4180 candidate channels were found through the semi-automated search method, with 1102 meeting the exclusion criteria (§2.3). N.B. If channels were found by both methods, these were attributed to the manual search method. This gave a preliminary dataset of 1632 candidate channels. In the later validation stage (§2.1 and §2.5), a further 11 candidate channels were excluded due to channel deletion or total content removal and 2 candidate channels were excluded due to duplicate channel IDs (attributed to change of channel names, during the semi-automated search period). The resulting dataset of 1619 chemistry YouTube channels is provided in tabulated form as electronic supplementary materials.

**Figure 2.**
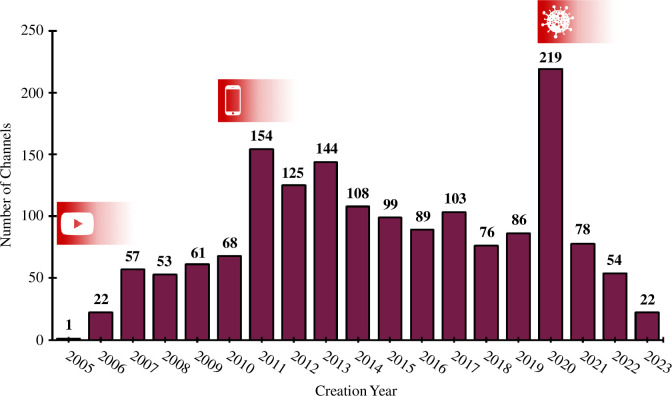
Number of channels created per year. Notable peaks occur in 2007, 2011 and 2020.

The number of chemistry YouTube channels created each year is shown in figure 2. There are years where there are peaks which are notably large compared to prior trends, i.e. 2007, 2011 and 2020. These can be correlated with the growth of YouTube [80], the proliferation of smartphones [81] and the outbreak of the COVID-19 global pandemic [82], respectively.

**Figure 3.**
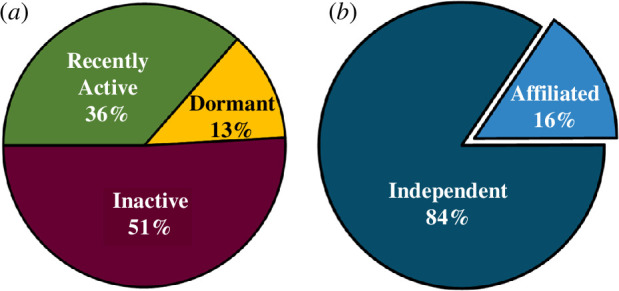
(***a***) Activity status of channels. (***b***) Proportion of channels that are independent versus those that display an affiliation. Channel affiliations can be found in table 4.

Regarding the activity of channels (see table 2 for definitions), 36% of chemistry YouTube channels were classified as ‘recently active’ during the sampling period (20 June 2023 to 1 August 2023). Thirteen per cent were ‘dormant’ and 51% were ‘inactive’ (figure 3*a*).

The majority of channels (84%) appeared to be independent (i.e. no clear affiliation), with only 16% of channels being clearly affiliated as per definitions in table 2 (figure 3*b* and table 4).

**Table 4.** Channel affiliations.

affiliation	percentage
private education provider	6%
university/college	4%
corporate	3%
learned society	1%
school	1%
other	1%
charity/non-profit	<1%

**Figure 4.**
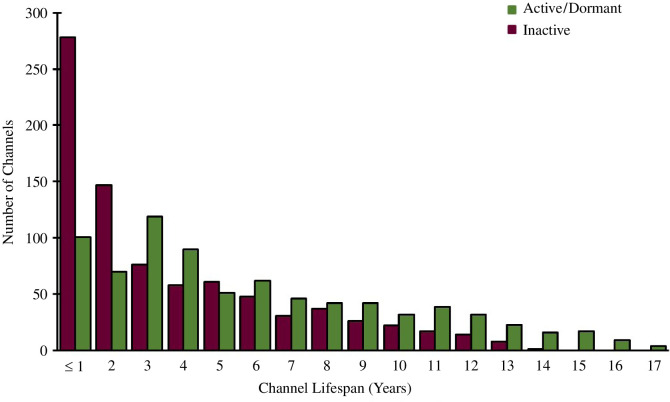
The lifespan of chemistry YouTube channels.

By calculating the timespan between the upload date of the first and latest video of each channel, we were able to determine a ‘lifespan’ for each channel which was further classified by active/dormant channels and inactive channels. There was no clear trend in lifespan for recently active or dormant channels. However, there was a clear exponential decrease in the lifespan of inactive channels with most inactive channels only producing videos in the lifespan of 1–2 years (figure 4). This pattern is similar to trends observed in the lifespan of science podcasts [4].

**Figure 5.**
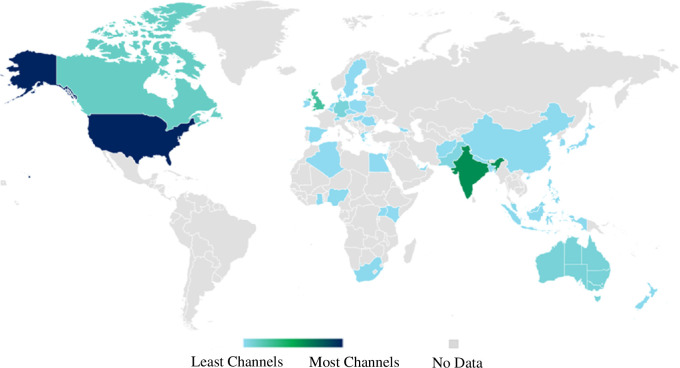
Global distribution of English-language chemistry YouTube channels.

Only 47% of channels listed a country of origin. Of these, the majority are from the USA and India, followed by the UK in third place (table 5 and figure 5).

**Table 5.** Top 10 countries producing chemistry YouTube channels.

rank	country	number of channels	percentage
1	USA	302	18.6%
2	India	184	11.4%
3	UK	78	4.8%
4	Canada	43	2.7%
5	Australia	25	1.5%
6	Germany	23	1.4%
7	Pakistan	16	<1%
8	Singapore	11	<1%
9	Malaysia	7	<1%
10	Netherlands	5	<1%
10	New Zealand	5	<1%
10	Philippines	5	<1%

**Figure 6.**
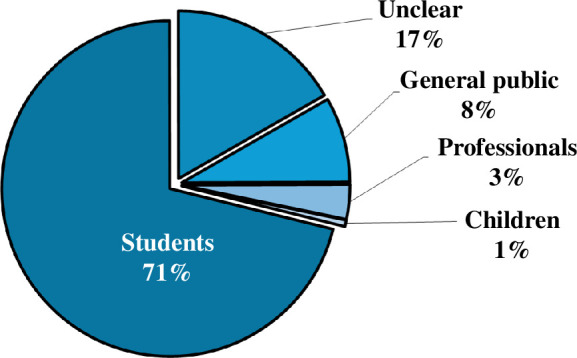
Overview of channels' perceived target audiences.

The largest apparent target audience for chemistry YouTube channels was students (71%) followed by the general public (8%). Three per cent of channels were found to be directed towards professionals, with less than 1% aimed towards children. Seventeen per cent of channels did not have a clear target audience (figure 6).

**Figure 7.**
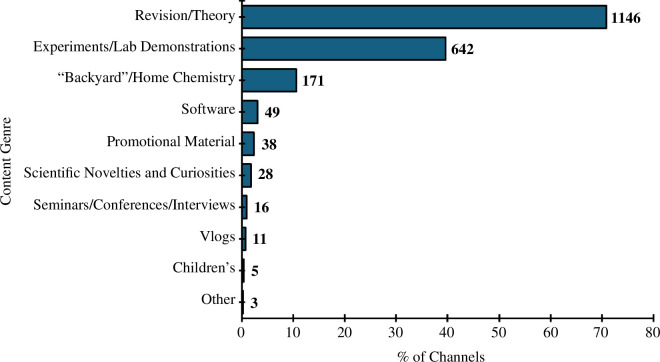
Summary of channel content genres. N.B. Channels may have spanned multiple genres, so percentages do not need to add up to 100%.

**Figure 8.**
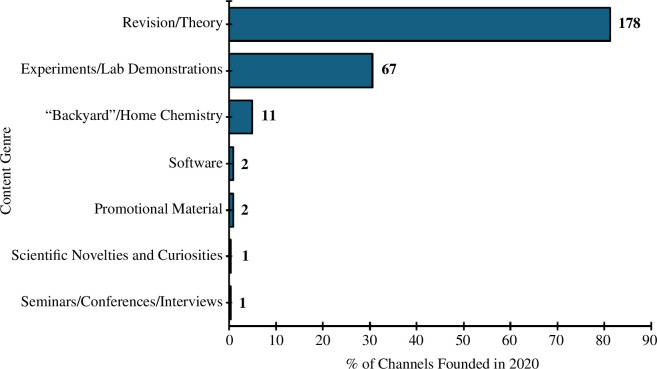
Summary of channel content genres for channels created in 2020. N.B. Channels may have spanned multiple genres, so percentages do not need to add up to 100%.

The most prevalent content genre was theory-related or revision videos (1146 channels; 71%). The next most prominent genre was experiments and laboratory demonstrations (642 channels; 40%), followed by ‘backyard’ and home chemistry (171 channels, 11%) (figure 7). Notably, the vast majority (81%) of channels created in the anomalous year of 2020 were focused on revision and/or theory content (figure 8).

**Figure 9.**
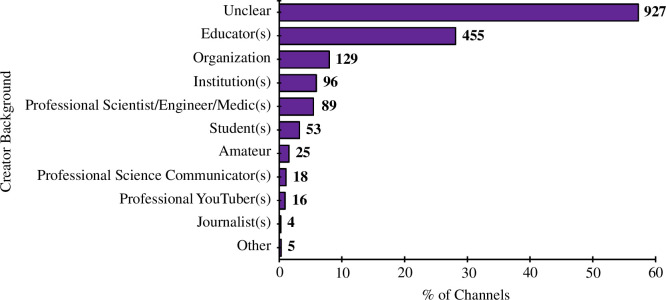
Background(s) of channel creator(s). N.B. Channels may have had multiple backgrounds, so percentages do not need to add up to 100%.

The background of the content creators was unclear for the majority of channels (57%). However, we found that there was a relatively large proportion of educators (28%), with 8% and 6% of channels also belonging to organizations and institutions, respectively. Two per cent of channels met the amateur categorization criteria (figure 9).

**Figure 10.**
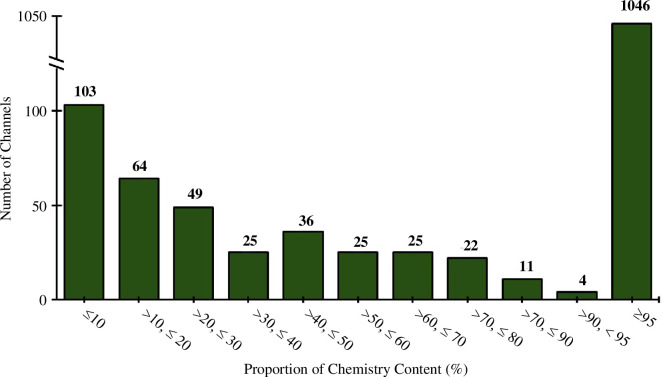
Estimated proportion of channels’ chemistry-focused content. Note the discontinuous *y*-axis.

The majority of channels were classified as ‘chemistry-only’ (>95% chemistry content) (*n* = 1046, 65%). The remaining ‘multi-disciplinary’ channels (*n* = 572, 35%) were those identified to have a proportion of chemistry content less than 95%. Two hundred and nine of these channels (13% of all channels, 37% of multi-disciplinary channels) could not be given a clear chemistry content proportion estimate, as they either did not present a dedicated chemistry playlist or were not selected for sampling. However, it was known from random sampling of every channel that they possessed a chemistry video ratio of less than 95%. We also found that chemistry is a minority content type for a large portion of multi-disciplinary channels, with 48% of the total multi-disciplinary channels (*n* = 277) having <50% chemistry content (figure 10).

**Figure 11.**
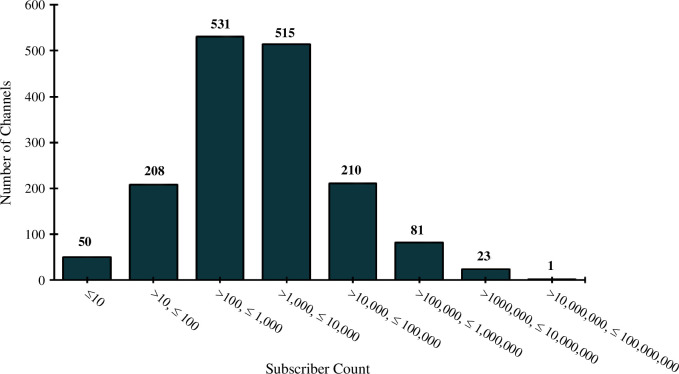
Channel subscriber count plotted on a logarithmic *x*-axis scale.

Subscriber count is an important metric which is a proxy for viewer interest in a channel. Seven hundred and eighty-nine channels (49%) had fewer than 1000 subscribers, 401 channels (25%) had between 1000 and 5000 subscribers, 323 (20%) had between 5000 and 100 000 subscribers, 81 (5%) had between 100 000 and 10 00 000 subscribers and only 24 (1.5%) had over 10 00 000 subscribers. A breakdown of this data can be found in electronic supplementary material, figure S1. When plotted on a logarithmic *x*-axis, it appears that subscriber count data resembles a lognormal distribution (figure 11).

**Figure 12.**
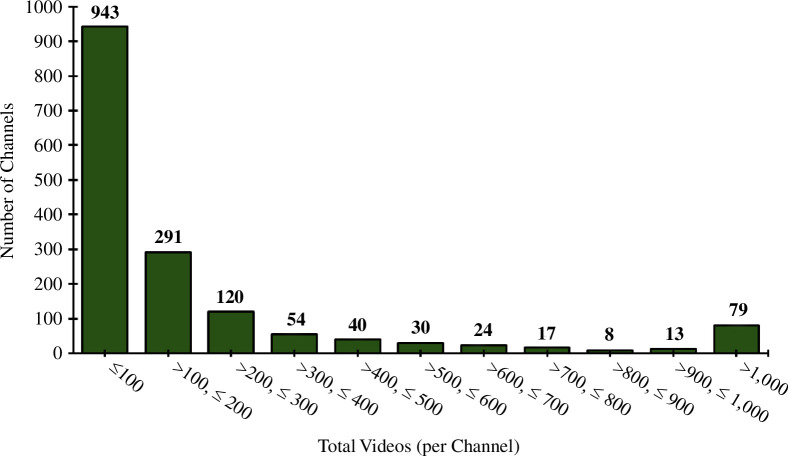
Number of videos uploaded per channel.

Regarding the number of videos produced by channels, we found that 943 channels (58%) had uploaded fewer than 100 videos, 597 channels (37%) had uploaded between 100 and 1000 videos and only 79 (5%) had posted over 1000 videos, with only 7 of those channels having a video count exceeding 10 000 (figure 12). A breakdown of this data can be found in electronic supplementary material, figure S2. This trend is perhaps unsurprising as it likely reflects the large amount of time, skill and effort required to start, maintain and grow a YouTube channel and release videos. In this dataset, there was no clear statistical correlation between the number of videos a channel had uploaded with the number of subscribers (0.206, Pearson product correlation coefficient) (electronic supplementary material, figure S3).

**Figure 13.**
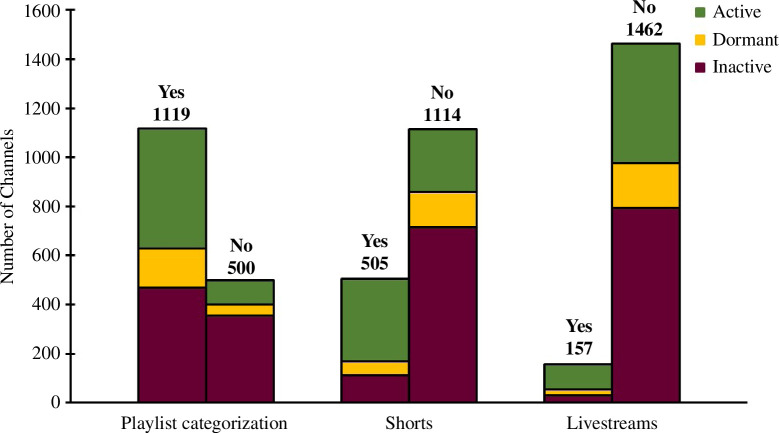
Utilization of playlists, shorts and livestreams by chemistry YouTube channels.

The majority of channels (69%) used playlists to organize content on their channels. A much lower proportion (31%) had uploaded ‘Shorts’ (i.e. short videos optimized for mobile format and social media sharing). Fewer still made use of YouTube’s capability for hosting video ‘livestreams’ (10%) (figure 13).

**Figure 14.**
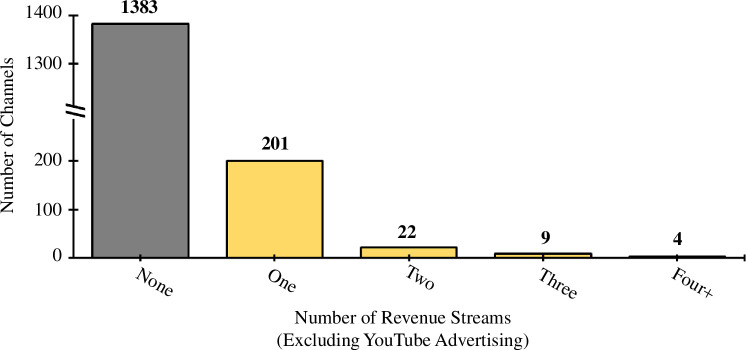
Number of channel revenue streams (excluding YouTube advertising).

**Figure 15.**
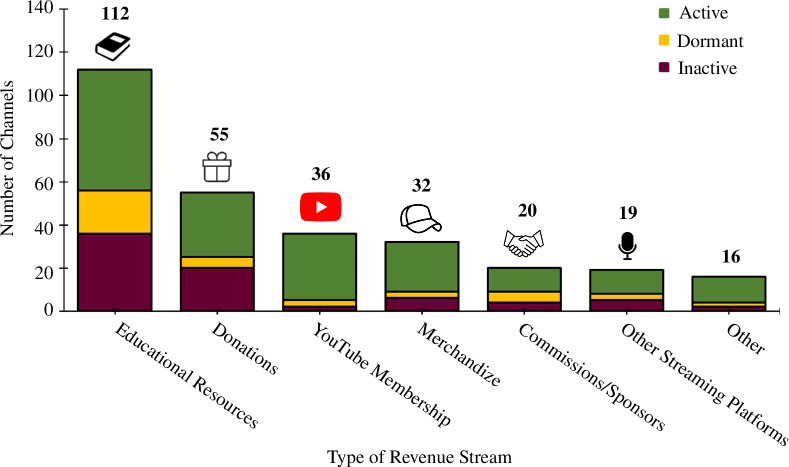
Breakdown of the various forms of additional channel revenue streams.

The vast majority of channels (85%) did not have any apparent additional revenue generation mechanisms, beyond the default YouTube advertisement revenue. Twelve per cent of channels had one additional revenue stream, with just over 2% providing details of two or more additional forms of revenue (figure 14). The most common forms of any additional revenue were educational resources, followed by donations, then YouTube membership (figure 15). However, our methodologies for assessing this had severe limitations (§4.1).

**Figure 16.**
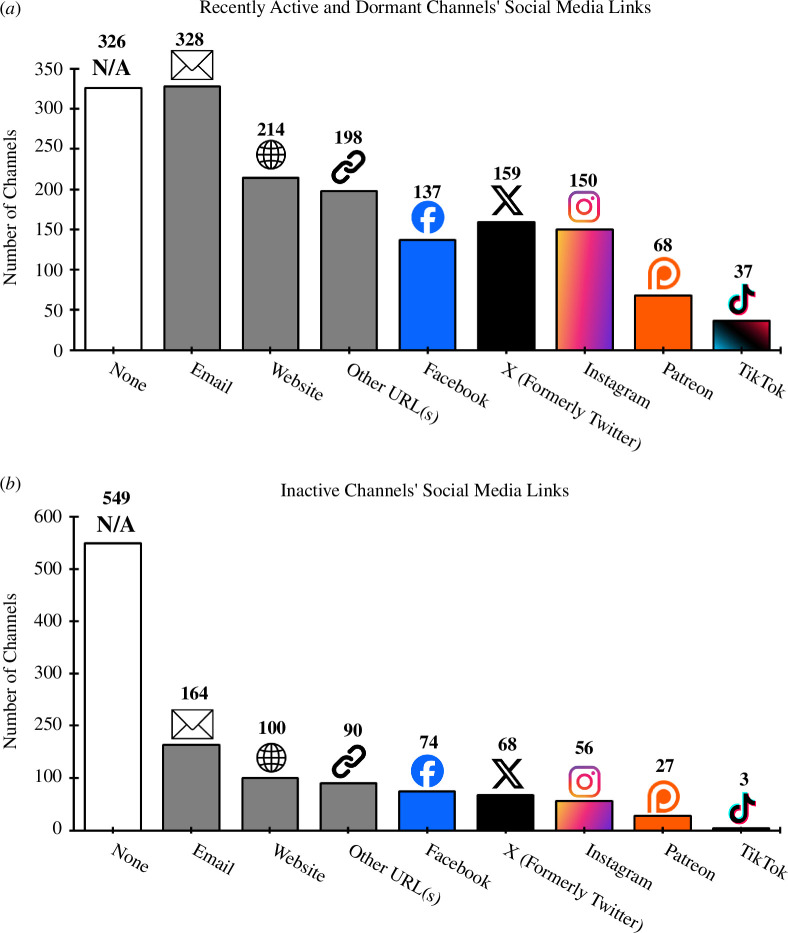
Overview of other social media links provided by (*a*) recently active and dormant channels and (*b*) inactive channels (see table 2 for definitions).

Regarding other social media, we found that 46% of channels provided links to one or more external websites and/or associated profiles, with email addresses and websites being the most popular types of links (figure 16). The most prevalent forms of other social media links were Facebook (founded in 2004) [83], X (formerly Twitter) (2006) [84] and Instagram (2010) [85] . Patreon (2013) [86] and TikTok (2016) [87] were the least common. Channels that were recently active or dormant (i.e. that had posted a video in the 12 months prior to sampling) were more likely to include all types of other social media links (figure 16*a*,*b*). Broadly it can be seen that the prevalence of each type of link is associated with the age of the given platform, as well as the given channel’s activity status.

## 4. Discussion

### 4.1. Methodology and associated limitations

Limitations of the quantitative methodology used must be acknowledged and recognized prior to further discussion of the results.

Firstly, only English-language YouTube channels were analysed, thus there is a paucity of data from non-English speaking regions of the world. Despite English-language-orientated search methods, a substantial number of non-English channels (*n* > 1127) were identified by the semi-automated search method and subsequently excluded from this study (figure 1). It is known that social media is utilized differently in different countries; as an unsubtle example, YouTube is banned in China [88], so there is likely to be minimal content on YouTube for Chinese audiences. Likewise, YouTube is banned in Iran [89]. These are two major countries with major scientific research programs. However, the use of virtual private networks can enable users in these countries to access YouTube [90]. Furthermore, news content presented through YouTube is more trusted in countries such as Brazil and India in comparison to the USA and the UK [91]. The prevalence of English-language chemistry YouTube content from India is not particularly surprising considering that India has a high degree of English speakers, has considerable scientific research interests, and has the largest population in the world of any country in 2024 [92,93]. As such, to ascertain a truly global snapshot of chemistry YouTube channels will require similar studies to be conducted in a variety of major international languages, such as Mandarin, Hindi, Russian, Spanish, Arabic and French, in order to better ascertain global trends in science communication.

Secondly, the content of YouTube videos was not utilized, other than to check if a channel met the English-language inclusion criteria. The channels analysed in this study had over 500 000 videos between them, so it would have been highly impractical to analyse these data through human means. Instead, the contextual information of a channel (i.e. its about page, website and other social media) along with channel metadata was used to glean insights. This approach was necessary for practicality, but has some notable shortcomings, for example, if a channel embedded adverts within videos (i.e. a sponsored segment), this would not have been apparent to our methodologies. This is particularly pertinent to our results for additional revenue incomes (figures 14 and 15), which likely under-represent revenue streams to some extent.

Thirdly, contextual data (such as perceived target audience) was categorized/coded by several individuals with chemistry backgrounds, in particular, three current undergraduate chemistry students (S. Gardner, G. Bezati and T. Godfrey) and a research staff member in a department of chemistry (L. MacKenzie). While this is significantly more robust than a single data coder and sufficient for this study, a larger panel of data coders would have been beneficial, particularly for improving the speed of post-acquisition data validation. Furthermore, there may be gaps between what the general public and university students/staff consider to be chemistry content. Additionally, there are categories of channel statistics that are not publicly available, instead privy only to the channel creator(s), such as average video watch time, advertising revenue, audience demographics, etc. Such data were reported for a chemistry YouTube channel (*ProfessorDaveatYork)* [94] in a study by Smith in 2014 [5]. It should therefore be acknowledged that the most accurate way to gain knowledge of chemistry YouTube content creators’ intent, insider knowledge and expertise would be to interview them. To date, such interviews have been conducted in limited numbers [95,96]. However, it would be impractical to arrange interviews for the large number of channels included in our study.

Fourthly, it should also be noted that due to the very large number of videos produced by the over 1600 channels included in this study, it was not practical to analyse whether or not videos used voice-over presentation style or if they had a visible presenter. Furthermore, it was not possible for us to analyse video comments. Such analysis of comments and presenters have been undertaken in smaller-scale studies, e.g. by Amarasekara and Grant (2018) [97], who also manually coded the gender of science video presenters.

### 4.2. Discussion of results and wider contextualization

The trends in chemistry YouTube channel activity can be compared to the comparable statistics for science podcasts gathered by MacKenzie in 2019, in which 46% of science podcasts had posted an episode in the three months prior to sampling [4]. For comparison, only 36% of chemistry YouTube channels had posted a video in the three months prior to sampling. This may be a result of two factors: (1) the relatively larger generic production burden of videos compared to audio-only podcasts; and (2) the fact that a large proportion of chemistry YouTube video channels are revision/theory-focused channels (figure 7), which may limit the content to a finite remit which can be fulfilled, thereby triggering the channel to stop releasing content.

The fact that the USA and UK are leading producers of English-language chemistry YouTube channels (table 5) is not surprising, and broadly correlates with viewership data published by a single chemistry channel case study by Smith in 2014 [5]. However, in this prior study, Smith did not note particular engagement with audiences from India, which we have identified as the second-largest country in terms of producing chemistry YouTube channels. Further research into target audiences and audience engagement of chemistry YouTube channels is needed.

Figure 6 shows that the largest apparent target audience for chemistry YouTube channels was students. This corresponds to figures 7 and 9, which show that large proportions of chemistry YouTube videos were structured around revision or theory content, and created by educators. Therefore, it is feasible that many students around the world are using YouTube as a free science learning resource, which has advantages over often relatively inaccessible materials, such as paid textbooks, tutors and personal contacts [1]. Indeed, several exploratory studies found in the existing literature indicate that YouTube is an effective and well-liked learning tool among chemistry students, with regard to both video consumption [98–100] and creation [5,101]. Consequently, it can be said that chemistry YouTube channels are emerging as a new paradigm for chemistry education as well as science communication more generally.

From figure 2, it can be seen that there are several years where the production of YouTube videos was very high compared to the proceeding and following years, i.e. 2007, 2011 and 2020. These years are associated with the growth of YouTube [80], the proliferation of smartphones [81] and the outbreak of the COVID-19 global pandemic and associated lockdowns [82], respectively. The increase in channels associated with the first two events could be described as a ‘natural progression’—as the popularity of YouTube itself grew, and access to the site became more ubiquitous, so did the number of users deciding to start creating their own content. The massive increase in the number of new channels in 2020 is a direct result of the global COVID-19 pandemic and consequential societal ‘lockdowns’ during 2020 and 2021. During this period, teachers and educators were more likely to post video content on video hosting platforms (of which YouTube is one). This is evidenced in our data, where it can be seen that a greater proportion of revision/theory-orientated chemistry YouTube channels were created in 2020 (figure 8) than in 2005–2023 overall (figure 7). More widely, during this period, people had time to take up creative pursuits [102], such as the creation of their own YouTube channels. Additionally, students used YouTube videos more substantially in the COVID-19 pandemic era than previously [103].

Our findings show that a large portion of chemistry YouTube content had been created by independent content creators with scientific backgrounds that are not made apparent to viewers (figures 3*b* and 9). This raises the question of whether or not the YouTube audience is concerned with the qualifications—or lack thereof—that a channel’s creator has. Again, this highlights the need for further study to understand the engagement of different chemistry video consumers with chemistry YouTube channels. For example, for those searching for study materials or experimental procedures, scientific accuracy is undoubtedly of paramount importance.

The advent of TikTok and short-form vertical video content has arguably set a new trend within the world of social media, proving itself an extremely engaging medium, particularly with regard to the ‘Generation Z’ age group [104]. A 2020 study by Hayes *et al*. noted that TikTok video content could increase public engagement with chemistry [105]. Likewise, a 2024 case study by Prindle *et al*. demonstrated a strategy to engage large public audiences with science [106]. A 2023 study by Graefen *et al*. found that educational content delivery through TikTok resulted in a statistically significant increase in student knowledge in the area of pharmacology, implicating TikTok as a potentially useful pedagogical tool [107]. However, a notable feature of TikTok in contrast to YouTube is that the videos TikTok users see are largely algorithmically recommended, rather than search-based. Therefore, if a TikTok viewer shows interest in science, they may be recommended a considerable amount of science videos that they would not have searched for otherwise. However, YouTube also includes recommendation features. Indeed, this propensity to similar content recommendation was used as a feature of our manual search procedure to identify chemistry YouTube channels, alongside a semi-automated search approach.

Some chemistry video content creators have both TikTok and YouTube channels, with a disparity in content and popularity between these video social media sites. A prominent example of a chemistry TikTok content creator (‘TikToker’) is Emmanuel Wallace (*Big Manny* [108]) who presents demonstrations on key chemistry topics such as alkali metals and chromatography. Big Manny has 1.8 million TikTok followers and just under 10 000 YouTube subscribers at the time of writing [109]. In 2021, YouTube introduced the aforementioned ‘Shorts’ feature to compete with TikTok [110], with other platforms such as Instagram and Facebook also introducing analogous features around the same time. Figure 13 shows that 505 channels included in this study (31%) had posted ‘Shorts’ content, and that these channels tended to be defined as recently active, likely reflecting that recently active channels are more up to date than inactive channels. It is an open question as to how the competition between short-form vertical videos and longer-form horizontal videos will transpire for science communication. To date, there has been limited exploration into the use of TikTok within the realm of science education and communication [111], therefore further research and new science communication research methodologies may be required to accommodate the rise of short-form TikTok-style vertical video content.

The limitations of our methodology (§4.1) meant that this study was not able to address important topics in science communication regarding the representation of diverse scientists as role models and trust in science. However, these issues should be noted and discussed. There is an ongoing need for diverse representation of scientists through all forms of media in order to challenge stereotypes. Multi-decade meta-studies have shown that scientists are stereotypically perceived as middle-aged Caucasian men, with this narrow stereotype discouraging engagement with science for those who do not fit this profile. Various approaches have been used to address these stereotypes, such as having scientists visit classrooms and the use of gender-equitable teaching materials [112–114]. Diversity in gender is an important factor in dispelling gender stereotypes. An analysis of 390 science YouTube videos by Welbourne & Grant [8] noted that the majority of science video presenters were male, and that this was consistent across both user-generated and professionally generated science YouTube videos [8]. However, it should be noted that women face barriers in creating YouTube content. In a study of 391 of the most popular STEM-themed YouTube channels, Amarasekara & Grant [97] noted a lack of female presenters, with a higher proportion of negative viewer comments, often with sexist themes [97]. This reflects the bias faced by female content creators online [97]. For a broader overview, readers are referred to Ferguson *et al.* (2020) [112]. Chen *et al.* (2021) have noted YouTube faces issues in that the most well-connected YouTube channels tend to have less diverse creators (in terms of racial and gender identities) [115].

Notably, YouTube is a visual medium where presenters can be seen, heard and related to by viewers. Thus, science YouTube channels are a potential medium that can help dispel harmful notions of stereotypes in science. For example, a study by Lee *et al.* [116] found that community college students who watched YouTube videos of prior college engineering students with similar backgrounds to the participants received higher engineering course grades and were more likely to enrol into an engineering course [116]. Diverse representation in science is also important because viewers engage with perceived authenticity and develop parasocial relationships with presenters, thereby engendering a positive bias and garnering more trust with specific creators or presenters [117]. This can be helpful in the wider sphere of science communication, e.g. in climate research, where misleading information is rife [118]. A prominent example of a trusted YouTube chemistry creator is the aforementioned Big Manny. Speaking with an East London Street accent combined with Jamaican patois and slang in his chemistry videos, Big Manny is seen as breaking down negative stereotypes. He has noted that his authentic style enables children to engage with science, who may not otherwise do so [119]. The value of such an authentic audience reach extends beyond science communication; highlighted by the UK Prime Minister’s Office collaboration with Big Manny in 2024, endorsing a video where he melted down a knife to highlight a ban on zombie knives in the UK [120,121]. This is a valuable tacit endorsement of both the scientific and cultural capital of such creators.

### 4.3. Open questions and opportunities

There are many aspects of chemistry and science YouTube videos which could be subjected to further investigation. For example, analysis of video titles [122,123], analysis of video thumbnail images [124,125] and sentiment analysis of video comments [97]. Machine-learning approaches could potentially be used to scale up the efficiency of such analyses [126,127], but this is still an area of ongoing development and significant discourse. Questions remain about how different audience demographics (age, background, location, language) find and engage with chemistry and science content, and if they trust this content. Furthermore, the extent of misinformation and conspiracy theories that pollute various areas of online science communication warrants significant investigation. Perhaps most prominently, it is also unclear how to best approach analysis of TikTok videos for understanding new and innovative forms of popular science communication. Therefore, new approaches to studying social media are required for future analysis of TikTok and content found on the platform.

Given our findings that YouTube chemistry channel creators are largely independent with limited means of financial support, organizations or individuals seeking to support diverse creators in chemistry and science on YouTube should consider how to support channel creators financially, in order to enable channels to grow in a manner that is authentic and encourages different audiences to engage with chemistry and science.

YouTube provides an opportunity to showcase hands-on chemistry techniques with all the ‘nitty gritty’ details provided either directly or within the context of the video. This is particularly powerful for teaching laboratories, with the Chemistry Teaching Labs at the University of York being an excellent example [128]. However, YouTube is also making an impact in the peer-reviewed chemistry literature as a credible source for synthetic chemistry experimental protocols with several notable examples to-date [11,12]. We suggest that creators should associate suitable chemistry YouTube videos to a digital object identifier (DOI) [129]. FigShare is one example of a platform which can be used to associate videos with DOIs [130]. This would assist in citing and discussing chemistry YouTube videos in the academic literature and help video creators gain credit for their work.

## 5. Conclusions

This study provides the first large-scale ‘census’ of chemistry content on YouTube. We used both manual and semi-automated approaches to identify 1619 YouTube channels producing English-language chemistry content. All data compiled in this study is provided as a tabulated database, which could be used for further in-depth data analysis or as a reference for future studies.

Our key findings are that there were several landmark years for chemistry YouTube channel creation, 2007, 2011 and 2020. These can be attributed to the rise of YouTube, the adoption of smartphones and the outbreak of the COVID-19 pandemic, respectively. Of the 1619 channels surveyed, 49% had posted a video in the 12 months prior to sampling. We found that a broad array of countries around the globe are producing English-language chemistry YouTube channels, but the USA, India and the UK are the most prevalent. We found there are various clear genres of chemistry YouTube content, and that the majority of chemistry YouTube channels are producing content focused on chemistry education and exam revision which is targeted to school or university-level students. Consequently, many channel creators have an educational background. We also found that a great deal of chemistry YouTube content is being produced by independent creators whose background is unclear, and who have no immediately apparent financial support mechanisms aside from YouTube advertising revenue. The majority of chemistry YouTube channels produced fewer than 100 videos.

Several open questions remain, including how different user demographic cohorts find and engage with science and chemistry content, and how algorithmic network effects may reinforce user interest and interaction with science and chemistry content on YouTube. Furthermore, it is unclear how different viewers may engage with chemistry and science YouTube content, how reliable they perceive the content to be and how much users trust this content. Crucially, the landscape of all forms of social media is constantly in flux [131]; this is well emphasized by the current rise in TikTok and associated science content. New approaches will have to be developed to identify and analyse chemistry and science content on current and future social media platforms in a timely manner.

## Data Availability

Supplementary material is available online [132].

## Footnotes

^1^
Subscribers are users which have ‘opted-in’ to receive content updates from a channel. Although it is not necessary to subscribe to a channel to watch its content, a channel’s subscriber count can be considered a proxy measure of a YouTube channel’s popularity.
